# Atypical Histoid Leprosy With Virchowian Features: A Report of a Histologically Deceptive Case

**DOI:** 10.7759/cureus.88017

**Published:** 2025-07-15

**Authors:** Azalea Guadalupe Altamirano De La Cruz, César Alberto Santoyo Reza, Francisco Javier Lugo Rincón-Gallardo, Karen Sofia Cruz Dominguez, Lizeth Nayeli Gutiérrez Gómez

**Affiliations:** 1 Internal Medicine, Hospital Dr. Jose Alonso A Diaz de León, Ensenada, MEX; 2 Internal Medicine, Instituto Mexicano del Seguro Social Hospital General de Zona No. 57, Estado de México, MEX; 3 Internal Medicine, General Hospital of the Institute for Social Security and Services for State Workers, Querétaro, MEX; 4 Internal Medicine, Institute for Social Security and Services for State Workers HG90, La Paz, MEX

**Keywords:** histoid leprosy, lepra, multibacillary, mycobacterium leprae, skin nodules

## Abstract

Histoid leprosy is an uncommon clinicopathological variant of Hansen’s disease, characterized by distinct immunological, clinical, histological, and bacteriological features. It is regarded as an intensified form of multibacillary leprosy, marked by an enhanced cellular and humoral immune response. Clinically, this condition typically presents with well-defined papules and nodules on otherwise normal-appearing skin, frequently located on the face, trunk, and extremities of the body. Histopathologically, it is marked by the proliferation of spindle-shaped histiocytes in a “whirl” or “storiform” pattern, accompanied by a significant presence of bacilli, often organized into “bacillary globi.” The importance of this variant lies in its high bacillary load, which makes it a potentially significant source of disease transmission if not diagnosed and treated promptly. This case report details the presentation of a 49-year-old male with a history of Hansen’s disease, who presented with widespread eucromic to violaceous papules and nodules across his face, upper extremities, torso, and back. Histopathological examination of a cutaneous biopsy revealed a Virchowian pattern, specifically a diffuse dermal infiltrate of foamy histiocytes with an exceptionally high bacillary load and a notable absence of the typical spindle-shaped histiocytes or storiform pattern usually associated with histoid leprosy. This atypical histological presentation, diverging from classic histoid morphology, underscores the critical importance of accurate diagnosis and appropriate treatment to limit its dissemination and prevent significant sequelae in patients. Through this case, we aim to highlight the histologically deceptive nature of some histoid leprosy presentations, emphasizing the fundamental importance of clinicopathological correlation for the accurate diagnosis of this neglected tropical disease and effective disease control.

## Introduction

Hansen’s disease, or leprosy, caused by *Mycobacterium leprae* (identified by Gerhard Henrik Armauer Hansen in the 19th century), is a chronic granulomatous mycobacteriosis [1-3]. This systemic disease primarily affects the skin, manifesting with diverse cutaneous symptoms, and is characterized by neurological involvement affecting peripheral nerves [3-5]. The immune response of the patient largely determines the clinical and histological presentations of leprosy. The World Health Organization (WHO) has outlined the following three cardinal diagnostic indicators: thickening of peripheral nerves, erythematous or hypopigmented skin lesions with sensory loss, and the presence of acid-fast bacilli in bacilloscopy or biopsy [2,4,6].

Histoid leprosy, first described by Wade in 1963 [1], is an uncommon morphological and clinicopathological variant within the broad clinical spectrum of lepromatous leprosy [2,6]. It is considered a more severe form of multibacillary leprosy, distinguished by unique immunological, clinical, histological, and microbiological features, including enhanced humoral and cellular immune responses [2,4,6]. Typically, histoid leprosy presents as well-defined papules and nodules on seemingly normal skin. Histopathologically, these lesions are characterized by a high bacillary count and spindle-shaped cells arranged in a storiform pattern, often accompanied by bacillary globi [2,7].

Despite significant advances in polychemotherapy, leprosy remains a global public health concern, with persistent new cases annually, indicating ongoing disease transmission [5,8]. Owing to its high bacillary burden, histoid leprosy serves as a substantial reservoir of infection, posing a major impediment to global elimination efforts [3,7]. The variable morphology of histoid leprosy frequently presents a diagnostic challenge, potentially delaying timely detection and appropriate treatment.

Furthermore, the transepidermal exit of *M. leprae* has been hypothesized to be a potential route for its transmission [3,9]. This clinical case aims to highlight an atypical manifestation of histoid leprosy, underscoring the critical importance of accurate diagnosis and appropriate therapeutic intervention to limit its dissemination and prevent significant patient sequelae.

## Case presentation

A 49-year-old male patient was referred due to nodular lesions present on his face, upper extremities, torso, and back, evolving over 15 months. He reported a history of Hansen’s disease four years prior but denied fever, weight loss, changes in sensation, or muscle weakness.

**Figure 1:**
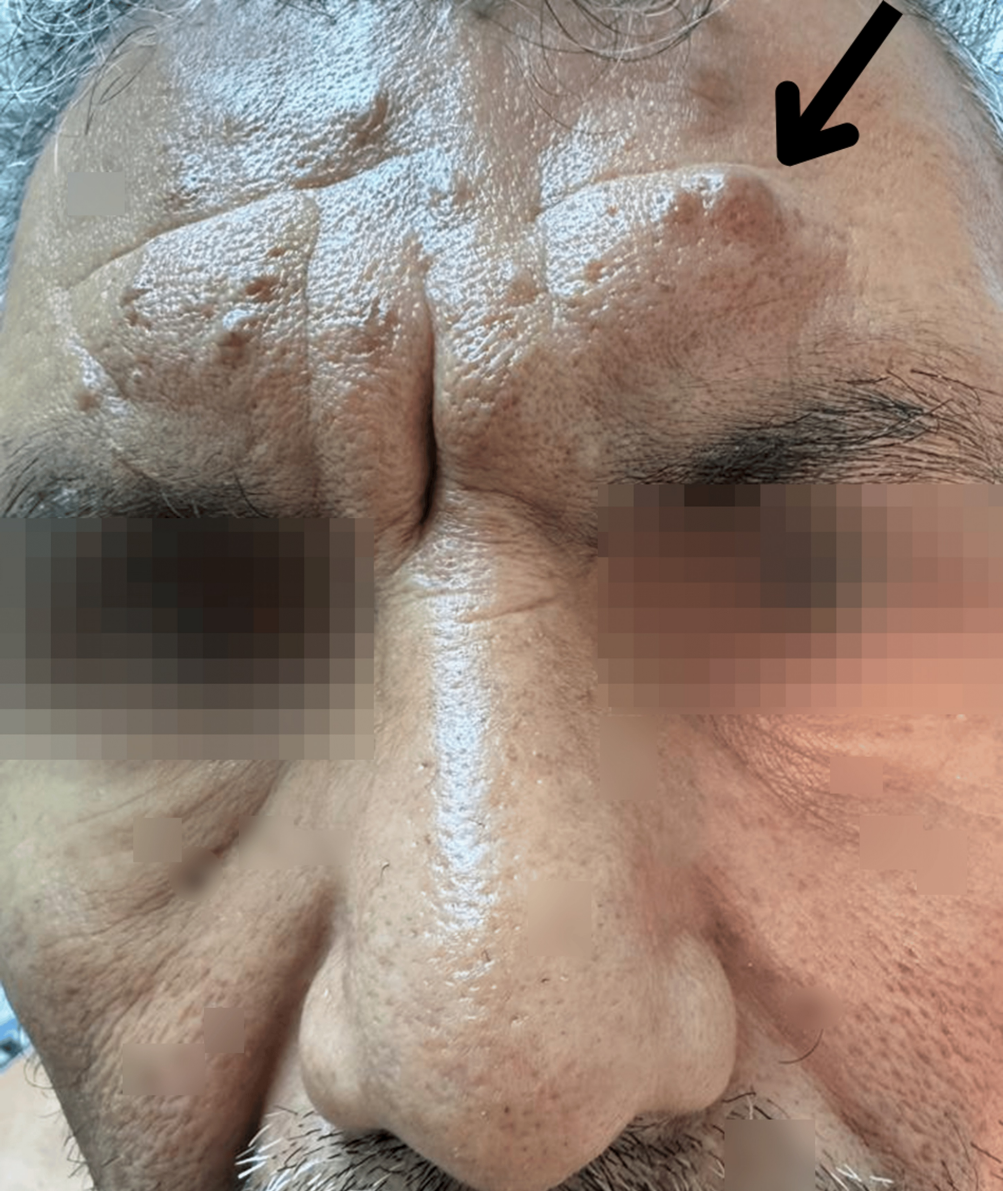
Facial dermatosis is evident, encompassing extensive involvement of the forehead and eyebrows. The frontal region, including the glabella, presents conspicuous nodules and significant cutaneous thickening. The arrow delineates areas of marked pathological involvement, where the skin appears substantially thickened and exhibits a clustered nodule or infiltrated plaque morphology.

**Figure 2:**
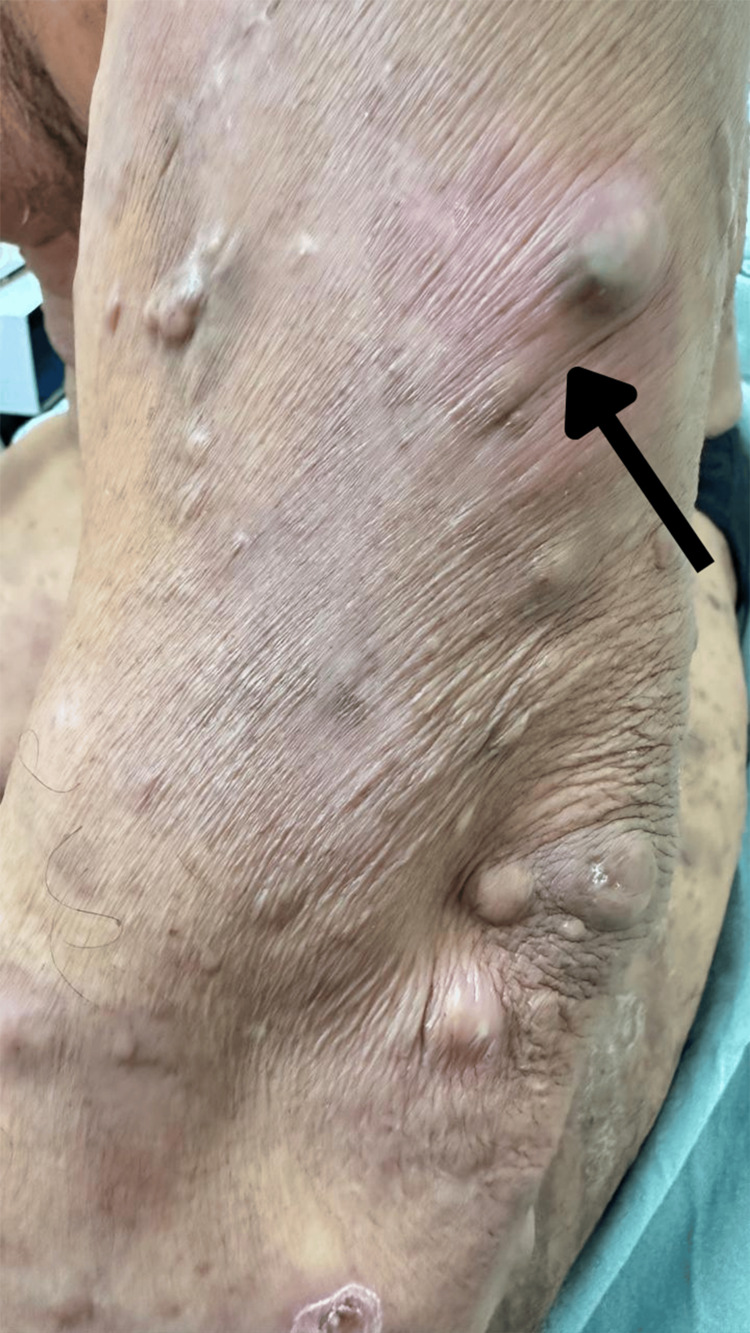
The upper extremity reveal dermatosis consisting of numerous subcutaneous nodules, predominantly normochromic compared to the surrounding integument, although some exhibit violaceous discoloration. A larger and more prominent nodule with a darker hue is distinctly visible and demarcated by an arrow. The overlying skin appears taut in the localized areas.

**Figure 3:**
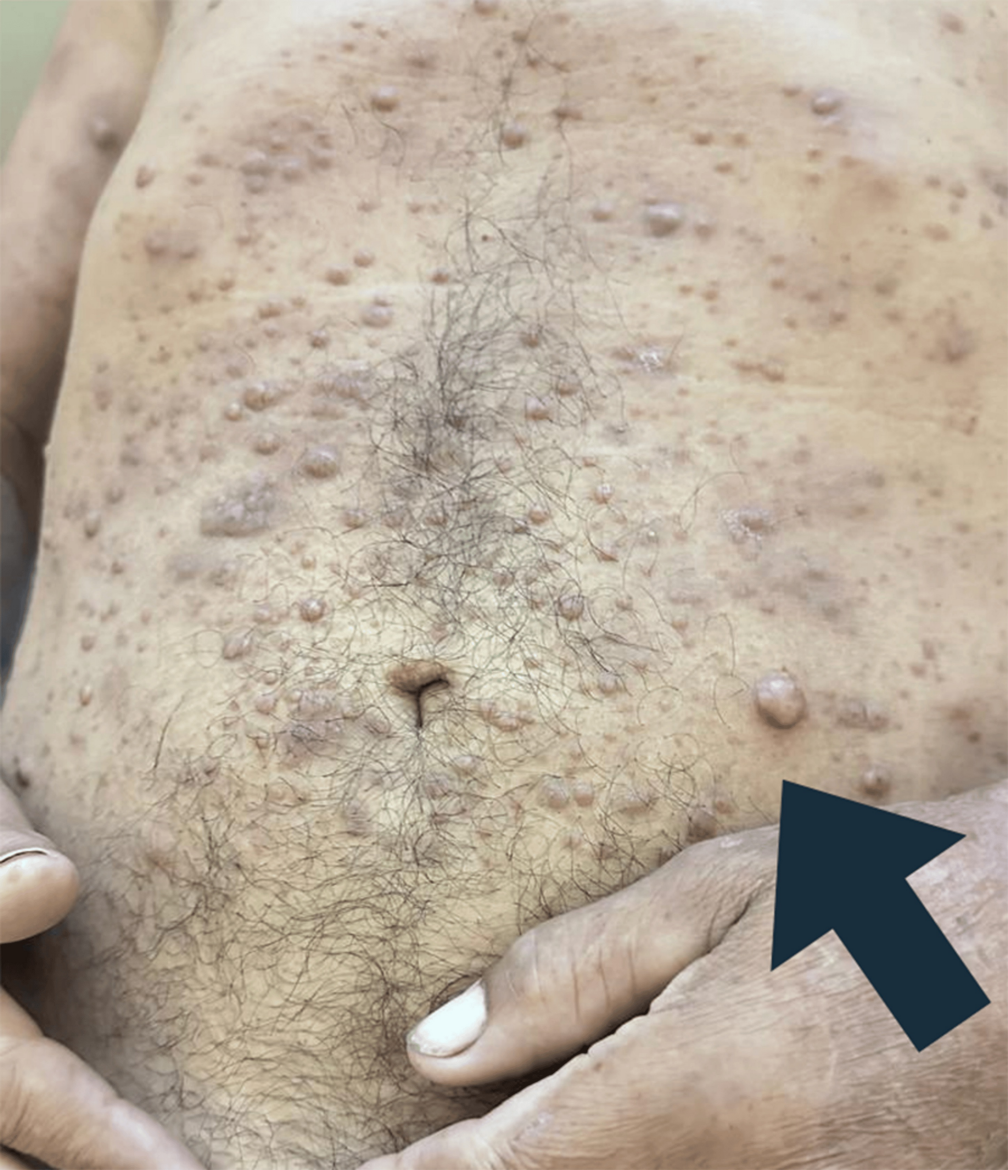
A magnified view of the abdomen revealing dense aggregation of small glistening papules and nodules. The surface morphology of these lesions varied, ranging from smooth to subtly irregular. The arrow indicates a specific region exhibiting more concentrated nodule clustering.

A physical examination revealed multiple normochromic papules and nodules. These lesions appeared infiltrated, varied in size (0.5 and 2 cm in diameter), and had a smooth surface. The skin color was normal in some areas, and hyperpigmented and erythematous violaceous in others (Figures 1-3).

Physical examination did not reveal thickened nerves, alterations in muscle strength, or changes in sensation in either the upper or lower limbs. Laboratory results showed no abnormalities (Table 1).

**Table 1:** Initial laboratory test.

Laboratory test	Patient’s result	Normal range
White blood cells	9.3 × 10^3^/µL	4.0–11.0 × 10^3^/µL
Hemoglobin	15.2 g/dL	13.5–17.5 g/dL
Hematocrit	45.40%	38.8–50.0%
Mean corpuscular volume	95 fL	80–100 fL
Platelets	343 × 10^3^/µL	150–450 × 10^3^/µL
Neutrophils (absolute)	5.3 × 10^3^/µL	1.8–7.7 × 10^3^/µL
Lymphocytes (absolute)	1.23 × 10^3^/µL	1.0–4.8 × 10^3^/µL
Monocytes (absolute)	0.82 × 10^3^/µL	0.70–1 × 10^3^/µL
Eosinophils (absolute)	0.6 × 10^3^/µL	0.00–0.5 × 10^3^/µL
Basophils (absolute)	0.35 × 10^3^/µL	0.20–1 × 10^3^/µL
Creatinine	0.85 mg/dL	0.7–1.3 mg/dL
Urea	32 mg/dL	20–40 mg/dL
Blood urea nitrogen	8.3 mg/dL	7–20 mg/dL

**Figure 4:**
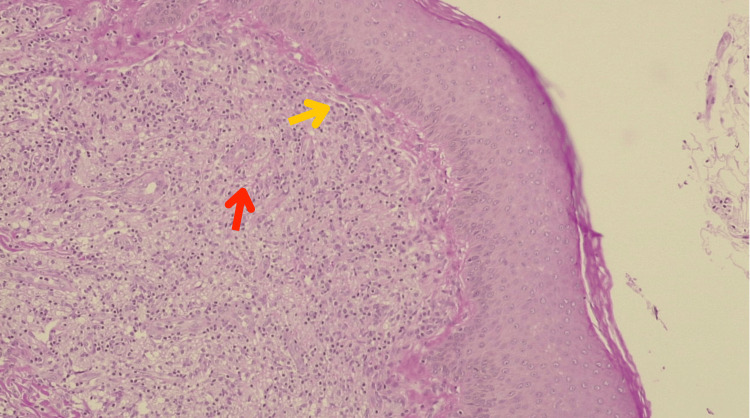
Histopathology (hematoxylin and eosin, 40×): A narrow band of normal or relatively unaffected dermal connective tissue can be seen immediately beneath the epidermis, separating it from a dense cellular infiltrate or underlying lesion in the dermis (yellow arrow). The dermis shows a diffuse dermal infiltrate composed of foamy histiocytes (red arrow). Bacilli were abundant, present not only intracellularly but also in an extracellular arrangement, a phenomenon reminiscent of the typical morphology seen in Virchowian lepromatous leprosy.

A cutaneous biopsy was performed. Hematoxylin and eosin staining revealed a Grenz zone. Furthermore, a diffuse dermal infiltrate composed of foamy histiocytes was identified, notably lacking nodule formation or fascicular organization, and without lymphocytes or granulomas. The bacillary load was extremely high, with abundant bacilli found not only intracellularly but also in an extracellular arrangement, a phenomenon reminiscent of the typical morphology seen in Virchowian lepromatous leprosy (Figure 4).

**Figure 5:**
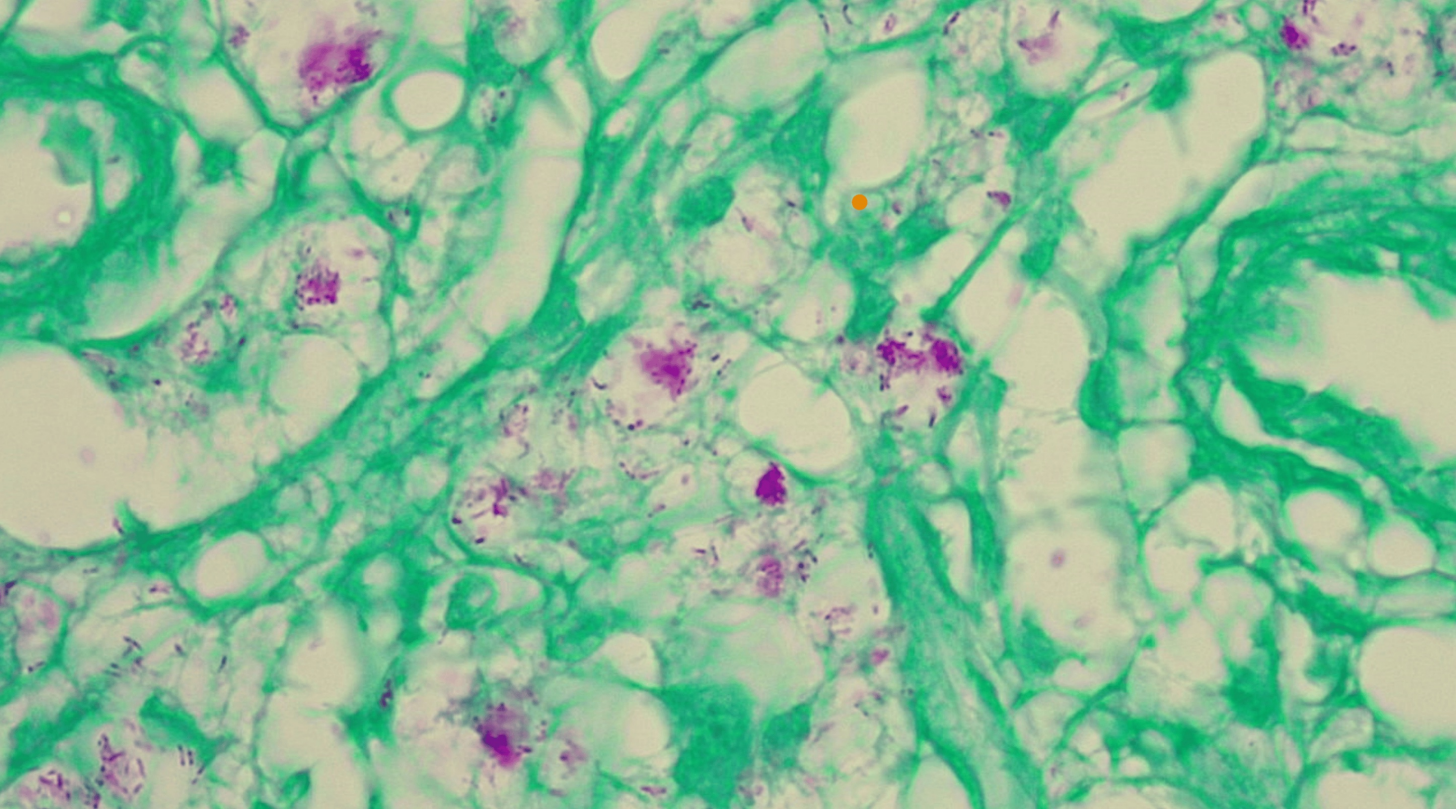
Histopathology (Ziehl-Neelsen staining, 100×): Abundant acid-fast bacilli and globi can be observed.

A cutaneous biopsy was also performed. Ziehl-Neelsen staining confirmed the presence of multiple bacilli, leading to the diagnosis of histoid leprosy (Figure 5).

Treatment was initiated with clofazimine, dapsone, and rifampicin for 12 months, accompanied by regular counseling and health education for the patient and his contacts to ensure treatment adherence.

## Discussion

Histoid leprosy is an uncommon yet critically important clinicopathological variant of multibacillary leprosy with significant epidemiological and clinical implications. Our patient, a 49-year-old male, precisely fits the predominant epidemiological profile for this condition, i.e., middle-aged adult males (40-50 years) with male-to-female ratios as high as 16:1 [2,3]. This demographic profile underscores the crucial need to consider this form of leprosy among this population. Although often associated with a history of lepromatous or multibacillary leprosy, or with partial or insufficient therapeutic regimens (e.g., dapsone monotherapy), histoid leprosy can also manifest spontaneously, appearing in up to 27.3% of cases [2,3,6]. The global persistence of leprosy, despite eradication efforts, is primarily attributed to inadequate socioeconomic conditions, host factors (such as immunology and genetics), and various environmental determinants [5-7,10]. The prevalence of histoid leprosy is increasing, particularly in India, where it is estimated to account for between 2.79% and 3.60% of all leprosy cases, with a growing incidence of de novo presentations [2,6,7].

Clinically, our patient presented with the characteristic features of histoid leprosy, namely, multiple euchromic papules and nodules, measuring 0.5 to 2 cm, which were distributed across the face, upper extremities, torso, and back, and developed over 15 months. Earlobe invasion is a distinctive sign of histoid leprosy, similar to that observed in lepromatous leprosy [2,5-7]. Histoid leprosy is recognized as a “great mimicker,” necessitating a high index of clinical suspicion to differentiate it from other dermatoses, including xanthomas, neurofibromas, dermatofibromas, reticulohistiocytosis, cutaneous metastases, acute sarcoidosis, keloids, and molluscum contagiosum [2,3,6].

Histopathologically, while histoid leprosy typically exhibits well-defined dermal or subcutaneous lesions with a subepidermal acellular zone (also known as the Grenz zone or Unna’s band) and an infiltrate of spindle-shaped histiocytes arranged in a storiform pattern, along with abundant bacilli (frequently grouped into “bacillary globi”) [2,6,7], our case demonstrated a notable deviation. Diffuse dermal infiltration of foamy histiocytes was observed, with a conspicuous absence of lymphocytes or granulomas, nodules, or fascicular architecture. This unusual presentation, combined with an exceptionally high bacillary load (Bacillary Index (BI) = 6+) and numerous intracellular and extracellular bacilli, more closely resembles Virchowian lepromatous leprosy with marked Th2 polarization [4,6]. This atypical histopathological manifestation, resembling Virchowian lepromatous leprosy rather than classic histoid morphology, aligns with recent reports detailing similarly challenging presentations and diverse histological expressions, including those with atypical cutaneous manifestations and transepidermal elimination of bacilli [10,11]. This particularity could be influenced by the host immunological status, genetic factors, or strain characteristics of the *M. leprae* strain [4,12].

Furthermore, the presence of bacilli at different epidermal levels reported in the literature [6,12,13] suggests upward migration and transepidermal elimination, which may be relevant in the dissemination of leprosy. Adverse reactions are common in histoid leprosy; erythema nodosum affects up to 40% of patients, a significantly higher percentage than other forms of the disease [2,14]. Peripheral neurological involvement is nearly ubiquitous, with thickening of two or more peripheral nerve trunks in most cases, and the ulnar nerve is most frequently affected [2,6,14].

The standard treatment for multibacillary leprosy, including histoid leprosy, follows the WHO multi-drug therapy regimen, consisting of rifampicin, clofazimine, and dapsone for at least one year [2,5,7]. This regimen was initiated in our patient, along with the necessary health education and follow-up. This case underscores the importance of considering leprosy in the differential diagnosis of widespread non-granulomatous dermal infiltrates, even when morphology deviates from typical patterns, particularly in endemic regions. This reaffirms the critical relevance of correlating histopathological findings with microbiological testing and a patient’s clinical presentation for accurate diagnosis and management, thereby minimizing the risk of misdiagnosis or delayed treatment, which could be considered a form of suboptimal care in patient care.

This case is limited by its single-patient design and absence of follow-up beyond the initiation of therapy. Additionally, immunohistochemical or molecular studies (e.g., polymerase chain reaction or cytokine profiling) were not performed, which could have provided insights into the immune dynamics or strain-specific features. Long-term monitoring of this patient and his contacts is essential for evaluating the clinical response and potential transmission.

Future studies should explore the immunogenetic basis of histoid presentations, particularly those with atypical histopathology. Studies focusing on cytokine expression, T-cell subsets, and genotypic profiling of *M. leprae* may help clarify the pathogenic spectrum of this disease.

By reporting this case, we aim to reinforce the critical role of clinical suspicion and histopathological confirmation, along with critical diagnostic tools such as slit skin smear for acid-fast bacilli, in the diagnosis of histoid leprosy, especially in atypical presentations. Our findings contribute to the understanding of this under-recognized form of Hansen’s disease and support continued vigilance in endemic areas.

## Conclusions

Histoid leprosy, an uncommon variant of Hansen’s disease, is characterized by a high bacillary burden and a substantial potential for community transmission. This case exemplifies the marked histopathological heterogeneity observed in this form of leprosy. The inherent diagnostic complexity of histoid leprosy, largely due to its ability to mimic various other dermatological conditions, underscores the critical importance of maintaining a high index of clinical suspicion, supported by rigorous clinicopathological correlation. To achieve effective and long-lasting epidemiological control, this case emphasizes the need for robust long-term follow-up protocols, which should include regular clinical re-evaluations, monitoring for drug efficacy and potential adverse reactions, and assessment of nerve function to guide the course of therapy. Additionally, implementing proactive early contact detection methods and sustained epidemiological surveillance systems is crucial to prevent further transmission. Continued research projects are also vital to elucidate the immunological and therapeutic complexities of this variant.

## References

[1] Wade HW (1963). The histoid variety of lepromatous leprosy. Int J Lepr.

[2] Gupta SK (2015). Histoid leprosy: review of the literature. Int J Dermatol.

[3] Canuto MJ, Yacoub CR, Trindade MA, Avancini J, Pagliari C, Sotto MN (2018). Histoid leprosy: clinical and histopathological analysis of patients in follow-up in University Clinical Hospital of endemic country. Int J Dermatol.

[4] Malhotra KP, Suvirya S, Malhotra HS, Kumar B, Gupta A (2019). Does histoid leprosy represent a locally hyperimmune variant of lepromatous leprosy?. QJM.

[5] Eichelmann K, González González SE, Salas-Alanis JC, Ocampo-Candiani J (2013). [Leprosy. An update: definition, pathogenesis, classification, diagnosis, and treatment]. Acta Dermosifiliogr.

[6] Mathur M, Jha A, Joshi R, Wagle R (2017). Histoid leprosy: a retrospective clinicopathological study from central Nepal. Int J Dermatol.

[7] Nair SP, Nanda Kumar G (2013). A clinical and histopathological study of histoid leprosy. Int J Dermatol.

[8] (2025). World Health Organization. Global leprosy (Hansen disease) update, 2021: moving towards interruption of transmission. https://www.who.int/publications/i/item/who-wer9736-429-450.

[9] Chen X, Liu HB, Shui TJ, Zha S (2021). Risk factors for physical disability in patients with leprosy disease in Yunnan, China: evidence from a retrospective observational study. PLoS Negl Trop Dis.

[10] Bathina A, Kollipara H, Sravani G, Laxmi VS (2024). Beyond classic leprosy: exploring atypical manifestations and their diagnostic challenges. Cureus.

[11] Babanrao SB, Tomar SS, Wankhade VH, Panindra L, Singh RP, Bhat D (2022). Histoid hansen's with transepidermal elimination: five cases. Int J Mycobacteriol.

[12] Vora RV, Pilani A (2014). Epidermotropism of lepra bacilli in a patient with histoid Hansen's disease. Indian Dermatol Online J.

[13] Alrehaili J (2023). Leprosy classification, clinical features, epidemiology, and host immunological responses: failure of eradication in 2023. Cureus.

[14] Kaur I, Dogra S, De D, Saikia UN (2009). Histoid leprosy: a retrospective study of 40 cases from India. Br J Dermatol.

